# Recent advances in the development of biocompatible nanocarriers and their cancer cell targeting efficiency in photodynamic therapy

**DOI:** 10.3389/fchem.2022.969809

**Published:** 2022-08-15

**Authors:** Sathish Sundar Dhilip Kumar, Heidi Abrahamse

**Affiliations:** Laser Research Centre, Faculty of Health Sciences, University of Johannesburg, Johannesburg, South Africa

**Keywords:** nanocarriers, photodynamic therapy, photosensitizers, targeted drug delivery, cancer, surface modification

## Abstract

In recent years, the role of biocompatible nanocarriers (BNs) and their cancer cell targeting efficiency in photodynamic therapy (PDT) holds potential benefits for cancer treatment. Biocompatible and biodegradable nanoparticles are successfully used as carrier molecules to deliver cancer drugs and photosensitizers due to their material safety in the drug delivery system. Biocompatible nanocarriers are non-toxic and ensure high-level biocompatibility with blood, cells, and physiological conditions. The physicochemical properties of BNs often enable them to modify their surface chemistry, which makes conjugating specific ligands or antibodies to achieve cancer cell targeting drug delivery in PDT. This review article focuses on the various types of BNs used in targeted drug delivery, physicochemical properties, and surface chemistry of BNs in targeted drug delivery, advantages of BNs in drug delivery systems, and the targeting efficiency of BNs on some specific targeting receptors for cancer therapy. Furthermore, the review briefly recaps the nanocarrier-based targeted approaches in cancer PDT.

## 1 Introduction

Cancer is a leading cause of death worldwide and the World Health Organization (WHO) reports that nearly 10 million deaths from cancer by 2020. According to the WHO report, the most common types of cancer-related deaths are breast, lung, colon and rectal, prostate, skin, and stomach cancers (WHO, 2022). Currently, several conventional cancer treatment options available including surgery, radiotherapy, and chemotherapy (Arruebo et al., 2011). As part of a cancer chemotherapeutic regimen, various anticancer drugs are used. Although these drugs have good cytotoxic properties, they also have acute side effects and cancer recurrence complications. The most common adverse effects of DNA-damaging drugs are occurred in the skin, gastrointestinal tract, and bone marrow (Weber, 2014). Debela et al., recently investigated new cancer treatment approaches and procedures, including stem cell therapy, targeted therapy, ablation therapy, gene therapy, and natural antioxidants (Debela et al., 2021). However, they have their own advantages and disadvantages when it comes to cancer treatments, and some of them have really supplied new potential for the benefit of cancer patients.

Nanocarrier-based drug delivery has been extensively studied in recent years, and it has shown promising results in cancer treatment. Nanocarrier-mediated drug delivery system protects loaded drugs from degradation, allows drugs to circulate in the bloodstream for longer periods, and efficiently targets cancer cells (Din et al., 2017; Wang et al., 2020). Nanoengineering or the fabrication of nanomaterial processes, will play a key role in designing functionalized nanocarriers with appropriate ligands to deliver the drugs to the target site (Patra et al., 2018). Several types of nanomaterials can be formulated for targeting cancer therapy which includes polymeric nanoparticles (Prabhu et al., 2014), liposomes (Riaz et al., 2016), dendrimers (Baher, 2009), inorganic nanomaterials (Sun et al., 2021), polymeric micelles (Oerlemans et al., 2010), hydrogel (Narayanaswamy and Torchilin, 2019), and carbon-based nanomaterials (Sajjadi et al., 2021). Wang et al., 2020 discussed the advancement of nanoparticle-based combination therapy and dual drug delivery, which facilitate achieving the synergistic potential of the drugs and overcoming drug resistance (Wang and Huang, 2020). Co-delivery of synergistic drugs using nanomaterials has more significant advantages such as drug protection, sustained drug release pattern, and enhancing drug mechanism of action in the target site (Afsharzadeh et al., 2017). Nanoparticle-based drug delivery systems can effectively deliver drugs using dual-targeting, and multiple targeting approaches for cancer cell targeting and imaging functions. Multiple targeting approaches are frequently used to improve therapeutic efficacy by targeting the subcellular molecules such as mitochondria, nucleus, and endoplasmic reticulum (Fu et al., 2020).

Photodynamic therapy has gained attention in cancer treatment in recent years owing to their benefits, such as the ability to kill cancer cells without causing any injury. In a nutshell, PDT is a minimally invasive cancer treatment that includes three main components: photosensitizers, light, and oxygen. Light-activated photosensitizers initiate a photochemical reaction that promotes the formation of reactive oxygen species, which can result in cell death via apoptosis or necrosis (Agostinis et al., 2011). Nanomedicine combined with photodynamic therapy can significantly improve the antitumor efficacy through immunogenic cell death (Jin et al., 2021). Park et al. recently discussed the limitations of clinically available nanomedicine for PDT. Poor water solubility, low yield of singlet oxygen quantum, high doses required to achieve therapeutic efficacy, and poor targeting efficiency on tumor site are some of the limitations of photosensitizers. The advancement of nanotechnology in drug delivery made it possible to overcome the limitations of photosensitizers for PDT therapy (Dabrowski and Arnaut, 2015; Park et al., 2021). Kumar et al., recently investigated the use of various biocompatible nanocarriers for cancer PDT applications, and it has shown positive outcomes in *in vitro* cell line studies and *in vivo* animal studies, as well as discussing the status of global clinical trials for various types of cancers in PDT (Kumar and Abrahamse, 2021). Li et al. recently discussed the development of next-generation nanomedicine delivery systems for targeted therapy, including stimuli-responsive systems for active targeting, tissue microenvironment reprogramming strategies, immunotherapy and transcytosable nanomedicine (Li and Kataoka, 2021). There is significant potential for using the above-mentioned next-generation delivery systems for PDT therapy to achieve more promising cancer treatment outcomes.

This review article explores the recent advances in the development of biocompatible nanocarriers and their targeting efficiency in cancer photodynamic therapy. Thus, this review article is divided into six different sections. Section 1 briefly explains the introduction to the study. Section 2 studied the different targeting approaches in cancer treatment. In Section 3, discussed the commonly used biocompatible nanocarriers in photodynamic cancer therapy and our primary focus is on polymeric nanocarriers, liposomes and dendrimers are discussed in detail. Section 4 describes the different types of targeting receptors for cancer therapy, and some of the recently reported nanocarrier targeted approaches to a cancer cell in PDT. In Section 5, future perspectives of this work are briefly discussed.

## 2 Targeting approaches in cancer treatment

A targeted drug delivery system is an emerging cancer treatment platform that protects healthy cells from cytotoxic cancer drugs, reduces dose-related adverse effects, and prevents the formation of drug-resistant cancer cells (Attia et al., 2019; Yao et al., 2020). In cancer treatment, the physicochemical properties of nanomaterials and the physiological conditions of the tumor microenvironment play an important role in drug delivery to the target region (Uthaman et al., 2018). The targeting approaches are classified into two types: passive targeting mechanisms and active targeting mechanisms. These two approaches offer the ideal solution for increasing delivered nanoparticle accumulation at the target site (Kleinstreuer et al., 2014).

### 2.1 Passive targeting mechanism

**FIGURE 1:**
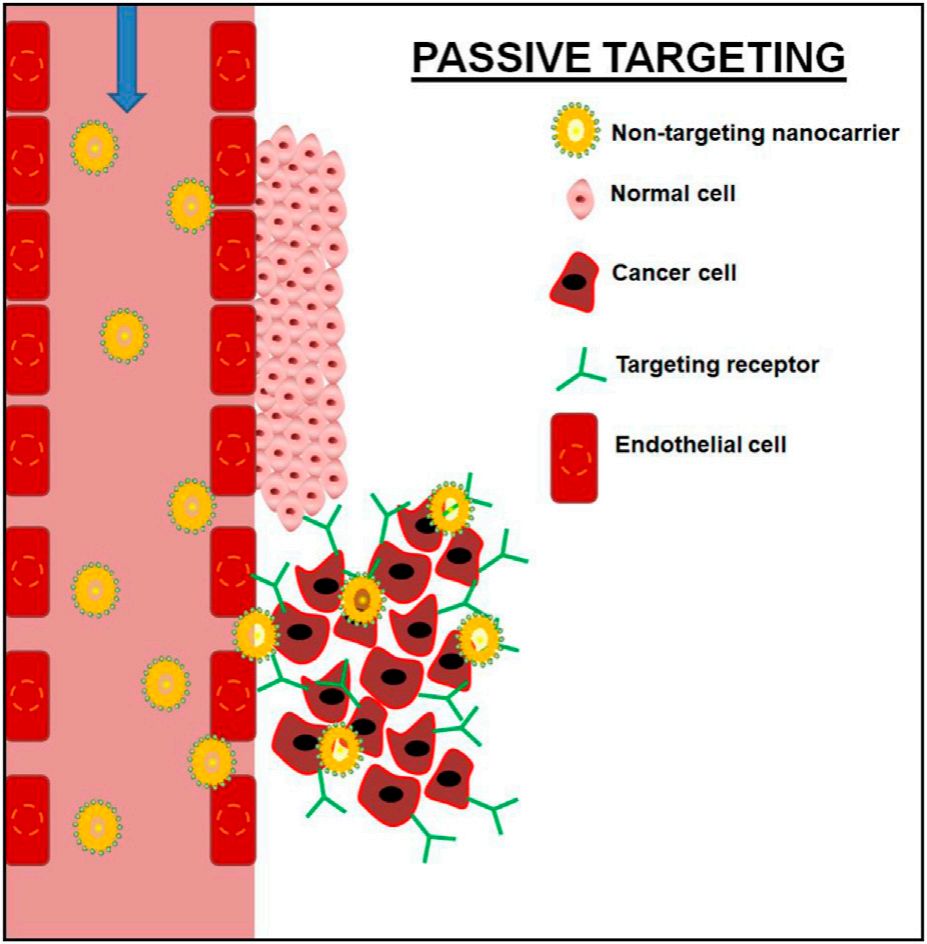
Schematic illustration of passive targeting of cancer cell using non-targeting nanocarrier.

Nano-sized materials with long systemic circulation properties frequently accumulate in the tumor site via a passive targeting mechanism which can be accomplished through an enhanced permeability and retention (EPR) effect. Nanomaterial accumulation in tumor cells via a passive targeting mechanism is entirely dependent on the permeability of leaky vasculature and decreased lymphatic drainage in tumor cells (Bazak et al., 2014; Nakamura et al., 2016; Wu, 2021). Figure 1 depicts schematic illustrations of passive targeting mechanisms.

Tsujimoto et al. (2021) synthesized fully polyethylene glycol-modified dendrimers with passive tumor targeting ability, and the hydration state of the dendrimer is important in drug carrier biodistribution. *In vivo* imaging revealed that the fully pegylated dendrimers accumulated in the tumor region of 8-week-old female balb/c mice injected with 4T1 (breast cancer cells). The presence of fully pegylated dendrimers in the tumor was also confirmed by *ex vivo* imaging results, as opposed to partly pegylated dendrimers. Several passively targeted nanomaterials are currently being tested in clinical trials for cancer treatment. Some of them are Doxil (a pegylated liposomal material for doxorubicin delivery, approved by FDA), DaunoXome (a liposomal material for daunorubicin delivery, approved by FDA), Marqibo (a liposomal material for vincristine sulfate delivery, approved by FDA), Onivyde or MM-398 (a pegylated liposomal material for Irinotecan delivery, approved by FDA), Abraxane (albumin-based nanoparticles for paclitaxel delivery, approved by FDA), Myocet (a liposomal material for doxorubicin delivery, approved in Europe and Canada), Mepact (a liposomal material for Muramyl tripeptide phosphatidyl-ethanolamine delivery, approved in Europe), SMANCS (a polymer conjugate for the delivery of neocarzinostatin, approved in Japan), and Genexol-PM (a polymeric micelle-based material for paclitaxel delivery, approved in Korea) (Shi et al., 2017).

### 2.2 Active targeting mechanisms

**FIGURE 2:**
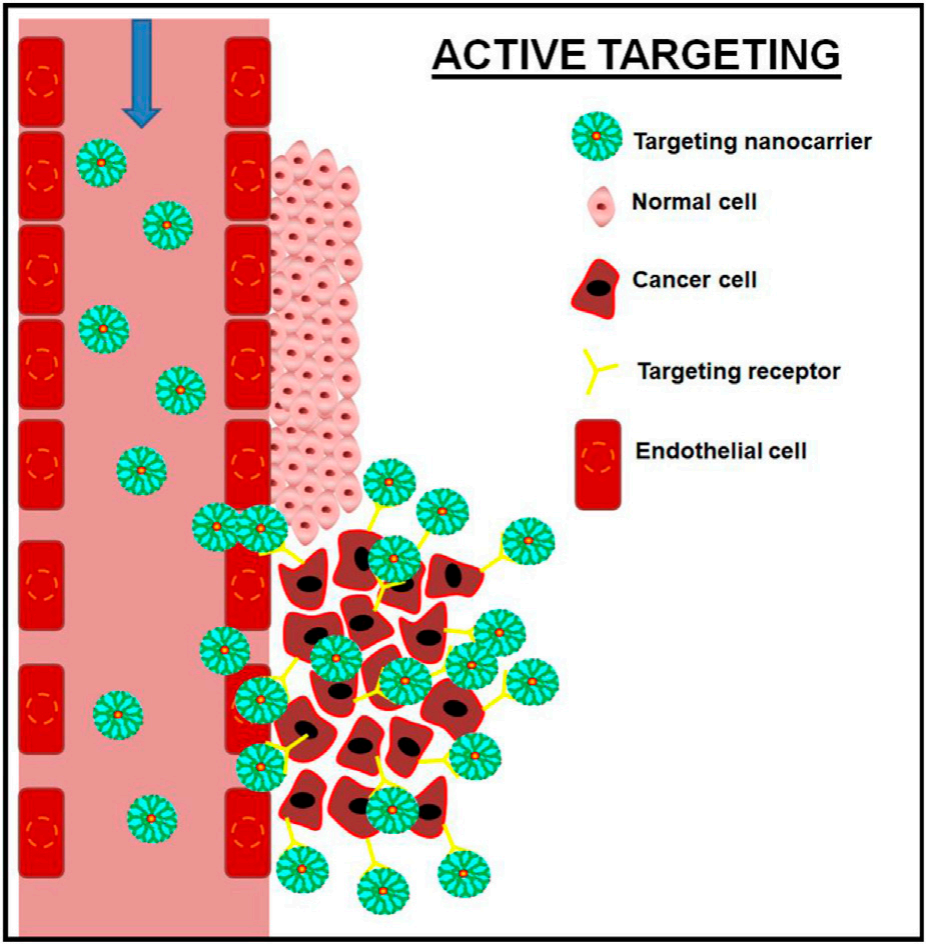
Schematic illustration of active targeting of cancer cell using targeting nanocarrier.

Selective targeting ligands/antibodies functionalized nanomaterials can target their appropriate receptors on cancer cells and facilitate the accumulation of active pharmaceutical ingredients in the tumor cell via an active targeting mechanism (Yoo et al., 2019). Figure 2 depicts schematic illustrations of active targeting mechanisms. Mehnath et al. (2020) synthesized and delivered paclitaxel for breast cancer treatment using active targeting polymeric micelles-based nanofibers, and it demonstrated high cytotoxicity against MCF-7 cells when compared to paclitaxel alone (Mehnath et al., 2020). Several active targeted nanomaterials are being tested in clinical trials for cancer treatment. Some of them are MM-302 (a HER2 receptor targeting liposomal material for doxorubicin delivery in Phase II/III clinical trial); BIND-014 (PSMA-targeting polymeric nanoparticles for docetaxel delivery in Phase II clinical trial); MBP-426 (a TfR targeting liposomal material for oxaliplatin delivery, in Phase I/II clinical trial); and Anti-EGFR immunoliposomes loaded with doxorubicin for solid tumors, in Phase I clinical trials (Shi et al., 2017).

## 3 Biocompatible nanocarriers in cancer photodynamic therapy

**FIGURE 3:**
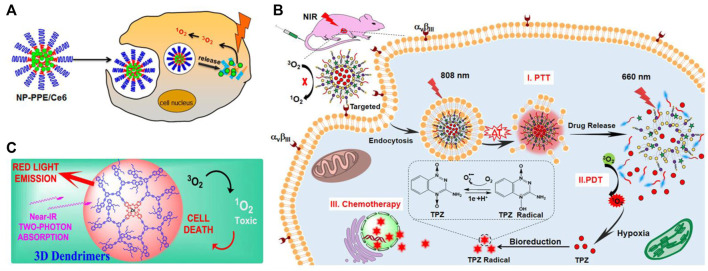
Schematic illustrations of biocompatible nanocarriers in cancer photodynamic therapy: **(A)** Polymeric nanoparticles—reprinted with permission from reference (Ding et al., 2015), Copyright 2015, American Chemical Society; **(B)** Liposomes—reprinted with permission from reference (Dai et al., 2019), Copyright 2019, American Chemical Society; and **(C)** Dendrimers—reprinted with permission from reference (Abid et al., 2021), Copyright 2021, American Chemical Society.

Biocompatible nanocarriers are widely used for various biomedical applications, including cancer PDT. Various types of biocompatible nanocarriers have been well reported for cancer PDT applications (Kumar and Abrahamse, 2021). This review focuses on three types of biocompatible nanocarriers that are commonly used for targeted approaches: polymeric nanoparticles, liposomes, and dendrimers. Figure 3 depicts schematic illustrations of biocompatible nanocarriers in cancer photodynamic therapy, and Table 1 discusses the various types of biocompatible nanocarriers used for cancer PDT in *in vitro* models.

**TABLE 1:** Different types of biocompatible nanocarriers used for cancer PDT in *vitro* models.

Name of the nanocarriers	Fabrication type	Size (nm)	Encapsulated compounds	Outcomes
Polyphosphoester Based Nanocarriers	Copolymerization	40 nm	Chlorin e6 (Ce6)	mPEG_45_-OH was used to synthesize Ce6-loaded polyphosphoester-based nanocarriers under the catalysis of 1,5,7-triazabicylo [4.4.0] dec-5-ene (TBD), followed by a dialysis method to remove unloaded Ce6. *In vitro* photodynamic therapeutic efficacy of synthesized nanomaterials was studied using BxPC-3 cells (pancreatic cancer cells) with and without NIR laser irradiation (660 nm), and it exhibited excellent cytotoxicity in irradiated treatment compared to free Ce6 Ding et al. (2015)
Polymeric micelles	Self-assembly	105.6 ± 28.56 nm	Doxorubicin and Ce6	The anticancer activity of doxorubicin-loaded polymeric micelles in 4T1 and A549 cells was investigated in *vitro*. In both irradiated and non-irradiated samples, laser irradiated, and non-irradiated dox-loaded micelles demonstrated increased activity compared to free dox Su et al. (2021)
Light-responsive polymeric nanoparticles	Emulsion-diffusion method	227.5 ± 5.1 nm	5,10,15,20-tetrakis (m-hydroxyphenyl) chlorine or (mTHPC)	The cytotoxic effects of mTHPC-loaded light-responsive nanoparticles on HT-29 cells (Colon cancer cells) were confirmed, and all formulations demonstrated an effective cellular uptake of mTHPC in fluorescence microscopy study Schoppa et al. (2021)
Multifunctional Theranostic Liposomes	Self-assembly	105 ± 5 nm	Indocyanine green (ICG); hypoxia-activated prodrug tirapazamine (TPZ) and Ce6	Multifunctional theranostic liposomes were used to coencapsulate ICG,TPZ, and Ce6, and their cytotoxicity in A549 cells (lung cancer cells) was confirmed. A flow cytometry study revealed significant cell apoptosis under 808 nm + 660 nm laser irradiation Dai et al. (2019)
Liposomes	Thin-film hydration method	From 99.6 nm ± 6.7–132.7 nm ± 12.7	Parietin	The parietin-loaded liposomes are synthesized in four different formulations. MDA-MB-231 cells (breast cancer cells) were used to test the cell viability of synthesized liposomes. The cell viability study confirmed the phototoxic effect of both free and drug-loaded liposomes in light and dose-dependent manner. A close examination of the study revealed that the photosensitizing activity of parietin was significantly increased when exposed to light Ayoub et al. (2021)
Co-encapsulated Liposomes	Coencapsulation	308 nm	Zinc phthalocyanine	MDA-MB 231 cells were used to test the cytotoxicity of coencapsulated liposomes. The study findings revealed that ZnPC-loaded liposomes significantly improved the cytotoxic effects of all concentrations tested Feuser et al. (2021)
Poly (amidoamine) PAMAM Dendrimers	Self-assembly	74.5 ± 18.4 nm	Ce6	U14 murine cervical cancer cells were used to study the cellular uptake and *in vitro* PDT efficacy of synthesized Ce6-loaded dendrimers. When compared to free Ce6, the PDT efficacy of Ce6-loaded dendrimers was significantly increased in the presence of H_2_O_2_ Xu et al. (2019)
Nanocrystal-dendrimer	Upconversion method	34 nm	Ce6	The cellular uptake of synthesized dendrimer was studied, and it demonstrated effective mitochondrial targeting in 4T1 cells (breast cancer cells) Liang et al. (2020)

### 3.1 Polymeric nanocarriers

Polymeric nanocarriers show great promise as a drug-loading molecule in cancer PDT (Li and Huh, 2014). It has a wide range of drug delivery applications, including high biocompatibility, surface functionalization, targeted drug delivery, high stability, biodegradation, and an interesting bio-mimetic character (Avramovic et al., 2020; Zhang et al., 2020). Polymer-based drug delivery is frequently used to achieve sustained and effective controlled release of loaded drugs at the site of action. The molecular structure of the polymer is important in the drug release mechanism. Furthermore, it improves the physicochemical properties of the loaded drugs indirectly (Liechty et al., 2010; Herdiana et al., 2022). Although polymeric nanocarriers have many advantages in drug delivery, they do have some disadvantages. Some of them include poor hydrophilic drug encapsulation, drug leakage before reaching target cells, the use of toxic solvents in the fabrication process, and the production of toxic monomers as byproducts of the degradation process (Kamaly et al., 2016). Polymeric nanocarriers can be prepared from either natural polymers or synthetic polymers. Natural polymers are biopolymers that are broadly classified into two types: proteins and polysaccharides. Chitosan, cyclodextrin, cellulose, hyaluronic acid, and starch are some of the natural polymers that are actively used as nanocarriers in cancer PDT. Synthetic polymer-based nanoparticles are widely used in drug delivery applications, including cancer PDT. Poly (D,L-lactic-co-glycolic acid) (PLGA), Poly-N-isopropylacrylamide (PNIPAAM), Polylactic acid (PLA) and polycaprolactone (PCL) are a few examples. In both *in vitro* and *in vivo* studies, natural and synthetic polymers demonstrated excellent biocompatibility and biodegradability (Sundar et al., 2016).

Porphyrins-loaded fluorescent polymeric nanoparticles were synthesized using the electrostatic interaction self-assembly method and were successfully tested for their dual function approach, including *in vivo* imaging and PDT effects. The synthesized nanoparticles are highly biocompatible and demonstrated excellent synergistic effects with 650 nm laser irradiation, such as tumor growth inhibition and fluorescence imaging-guided phototherapy against CT-26 tumor-bearing mice (Hou et al., 2022). Cong et al., synthesized 200 nm-sized near-infrared cationic polymer-based nanoparticles using the ring-opening polymerization method. An *in vivo* PDT effects of synthesized nanoparticles + light were studied using a 4T1-bearing nude mice model, and the study confirmed the effective inhibition of tumor size compared to the control, PBS with light, and the nanoparticles without light (Cong et al., 2022). Biodegradable silicon naphthalocyanine (SiNc) nanoparticles have been synthesized for use in bioimaging and cancer phototherapy. The nanoparticles were intravenously injected into the A2780/AD tumor-bearing nude mice, and the results showed that the tumors were completely eradicated in 3–5 days, with no recurrence of tumors detected (Taratula et al., 2015). *In vivo* photodynamic therapeutic effect of Chlorin e6 (Ce6) loaded nanomaterials were tested in BcPC-3 tumor-bearing mice. The synthesized Ce6-loaded nanomaterials were intravenously injected into tumor-bearing mice, and NIR light (660 nm, 0.5 W/cm^2^) was used to irradiate tumors; control mice were kept without laser irradiation. It has been confirmed that Ce6-loaded nanoparticles and NIR irradiation significantly inhibited tumor cell proliferation and apoptosis in tumor tissues (Ding et al., 2015). Li et al., synthesized chlorin e6 loaded hydrogen peroxide and poly (amidoamine) coassembled with triblock copolymer based polymeric vesicles for effective PDT treatment for hypopxic tumor and these nanocarriers not only facilitate delivery of photosensitizer but also have other functions like hypoxia alleviation (Li et al., 2017).

### 3.2 Liposomes

Liposomes are non-toxic, biodegradable spherical vesicles composed of a phospholipid bilayer, and it offers a promising carrier molecule in drug delivery (Akbarzadeh et al., 2013). Liposomes improve bioavailability and reduce the toxicity of loaded active pharmaceutical ingredients. Some of the pH-responsive liposomes provide numerous benefits in site-specific drug release (Lee and Thompson, 2017). Liposomes are commonly used as a carrier molecule for the delivery of photosensitizers in cancer PDT applications (Moghassemi et al., 2021). Liposomes have some drawbacks in drug delivery, including poor stability, low solubility, a short half-life, drug leakage and high production costs (Leitgeb et al., 2020).

In A549 tumor-bearing nude mice, the therapeutic efficacy of Indocyanine green (ICG), hypoxia-activated prodrug tirapazamine (TPZ), and Ce6-loaded multifunctional theranostic liposomes was investigated. Time-dependent fluorescence imaging studies confirmed that synthesized liposomes were actively targeting and accumulated in the tumor region. An *in vivo* activation of PDT and immunofluorescence staining study demonstrated that laser irradiation increases strong hypoxic signals due to the continuous oxygen consumption, and this leads to the activation of TPZ, facilitating achieving tumor-selective combination therapy (Dai et al., 2019). The synthesized long-circulating liposomes were injected intravenously into Meth-A sarcoma-bearing mice and irradiated using the following laser conditions: 689 nm laser, 150 J/cm^2^, 0.25W, and the study results show that the tumor growth was strongly suppressed (Ichikawa et al., 2005). Zhang et al., synthesized a novel liposome encapsulated with Ce6, Tirapazamine and gene probe for photodynamic and chemotherapy applications. An *in vivo* study was conducted using MCF-7 cells injected into a nude mouse, and the mice were then allowed to develop ∼75 mm^3^ tumors. After 4 h, the nanoparticle was injected into tumor-bearing mice and exposed to laser irradiation (670 nm laser). The results of the study revealed that the tumor was almost completely eliminated when compared to the control, indicating the synergistic effects of synthesized liposomes (Zhang et al., 2018).

### 3.3 Dendrimers

Dendrimers are multifunctional, hyperbranched polymers that have the potential to be used as functional materials in cancer photomedicine. It has a wide range of biological applications, such as theranostics, combination therapy, molecular imaging, and PDT/PTT (Ouyang et al., 2021). Dendrimer-based nanocarriers have shown promise in protecting the nature of phototherapeutic drugs in PDT applications. Dendrimer’s structural advantages enable easier surface modification of targeting ligands or functional moieties for targeted cancer therapy, and it improves therapeutic compound accumulation in the targeted site (Gupta et al., 2010; Zhu et al., 2019; Gao et al., 2020). At the same time, dendrimers have their own limitations in terms of proven toxicity issues, but there are several approaches, such as surface modifications using biocompatible materials, that can overcome dendrimer limitations (Chis et al., 2020).

Xu et al. (2019) investigated the efficacy of Ce6-loaded dendrimers in U14 tumor-bearing nude mice with the following *in vivo* PDT conditions: 670 nm laser (10 mW/cm^2^) for 30 min. An *in vivo* fluorescence imaging study revealed that Ce6-loaded dendrimers (with MnO_2_) completely inhibited tumor growth in mice when compared to free Ce6. Nishiyama et al., synthesized a dendrimer phthalocyanine encapsulated polymeric micelle for improved photodynamic cancer therapy. *In vitro* photocytotoxicity of a dendrimer-based polymer micelle was tested against A549 cells after laser irradiation using 670 nm diode laser, and it demonstrated fluence-rate dependent phototoxicity and induced photodamage in the mitochondria. An *in vivo* animal experiment was carried out using A549 lung cancer cells bearing mice, and the study results show the effective antitumor activity compared to the clinically used Photofrin (Nishiyama et al., 2009). Kojima et al., prepared rosebengal (RB) and protoporphyrin IX (PpIX) encapsulated in pegylated poly (amido amine) and poly (propylene imine) for PDT applications. The results of an *in vitro* cytotoxicity study on HeLa cells confirmed that RB alone and RB loaded dendrimers were equally toxic. Similar results were obtained in protoporphyrin IX-loaded dendrimers, confirming the easy release pattern of photosensitizers from dendrimers. Furthermore, the study found that hydrophilic RB releases faster than hydrophobic PpIX in PBS (Kojima et al., 2007).

## 4 Different types of targeting receptors for cancer therapy

Currently, there are several types of targeting receptors or ligands are currently available for nanocarrier-based targeted cancer therapy. Here are some examples: folate receptor, asialoglycoprotein receptor, CD 44 receptor, Her2—Herceptin receptor, CD 20 antigen; CD 19 antigen, EGFR receptor, CD 326 receptor, VEGF receptor, transferrin receptor, luteinizing hormone-releasing hormone receptor, and αvβ3 receptor (Yu et al., 2010). Table 2 lists the most commonly used targeting ligands and receptors for nanocarrier-based drug delivery systems in cancer therapy.

**TABLE 2:** Commonly used targeting ligands and targeting receptor for targeted cancer nanomedicine.

Name of the nanocarriers	Targeting ligands (small molecules, peptides, aptamers, proteins, antibodies)	Targeting receptor	Name of the cell line used	Types of cancer
Cis-diamine platinum drug and siRNA-loaded dendrimer-based nanoparticles	Folic acid	Folate receptor-α	H1299 - Lung cancer cells	Lung cancer Amreddy et al. (2018)
Gum kondagogu capped gold nanoparticles	Folic acid	Folate receptor	MCF-7 - Breast cancer cells	Breast cancer Kumar et al. (2018)
Doxorubicin-loaded pullulan-based nanocarriers	Arabinogalactan (a galactose-based polymer) and pullulan (a glucose-based polymer)	Asialoglycoprotein receptor (ASGPR)	HepG2 - Human hepatocellular carcinoma cells	Liver cancer (Pranatharthiharan et al., 2017)
Curcumin and doxorubicin-loaded hyaluronic acid-based nanoparticles	Hyaluronic acid	CD 44 receptor	A549 - Human non-small lung cancer cells; HepG2 - Human hepatocellular carcinoma cells	Lung and Liver cancer Diao et al. (2019)
Docetaxel loaded Trastuzumab-Coated Nanoparticles	Trastuzumab (Herceptin)	Human epidermal growth factor receptor 2 (HER2)	BT474—Breast cancer cells	Breast cancer Zhang et al. (2019)
Doxorubicin loaded liposomal-based nanocarrier	Human anti-CD20 monoclonal antibody	B-lymphocytes	CD20-positive Raji cells derived from Burkitt’s lymphoma	B cell lymphoma (Jiang et al., 2016)
Doxorubicin encapsulated polymeric nanoparticles	Biotinylated mouse anti-human CD19Ab	CD 19 receptor	RS4; 11 and REH cells - Human acute leukemia cell lines	Acute Lymphoblastic Leukemia (ALL) Krishnan et al. (2015)
Doxorubicin-loaded silver-coated gold nanorods	Epithelial cell adhesion/activating molecule	Epithelial cell adhesion/activating molecule (EpCAM receptor; CD326)	4T1—Breast cancer cells	Breast cancer (Jenkins et al., 2017)
Cetuximab conjugated temozolomide-loaded poly (lactic-co-glycolic acid) nanoparticles	Cetuximab	EGFR receptor	U-87MG - a human glioma cell lines	Glioblastomas Duwa et al. (2020)
Doxorubicin loaded bevacizumab modified nanoparticles	Bevacizumab antibody	VEGF receptor	SH-SY5Y - Human neuroblastoma cell line	Neuroblastoma Zhu et al. (2017)
Gold nanoparticles	Transferring peptide (Tf_pep_)	Transferrin receptor	U87 - Human glioma cells; LN229—glioblastoma cells	Brain tumors Dixit et al. (2015)
Iron Oxide nanoparticles	LHRH-R peptide and uPAR peptide	Luteinizing hormone-releasing hormone receptor [LHRH-R] and Urokinase-type plasminogen activator receptor [uPAR]	PC-3—Human prostate carcinoma cells	Prostate cancer (Ahmed et al., 2017)
Solid lipid nanoparticles	cyclic arginyl-glycyl-aspartic acid (cRGD) peptides	αvβ3 integrin receptor	MDA-MB-231 - human triple-negative breast cancer cell line	Breast cancer Shan et al. (2015)

### 4.1 Nanocarrier-based targeted approaches in cancer PDT

**FIGURE 4:**
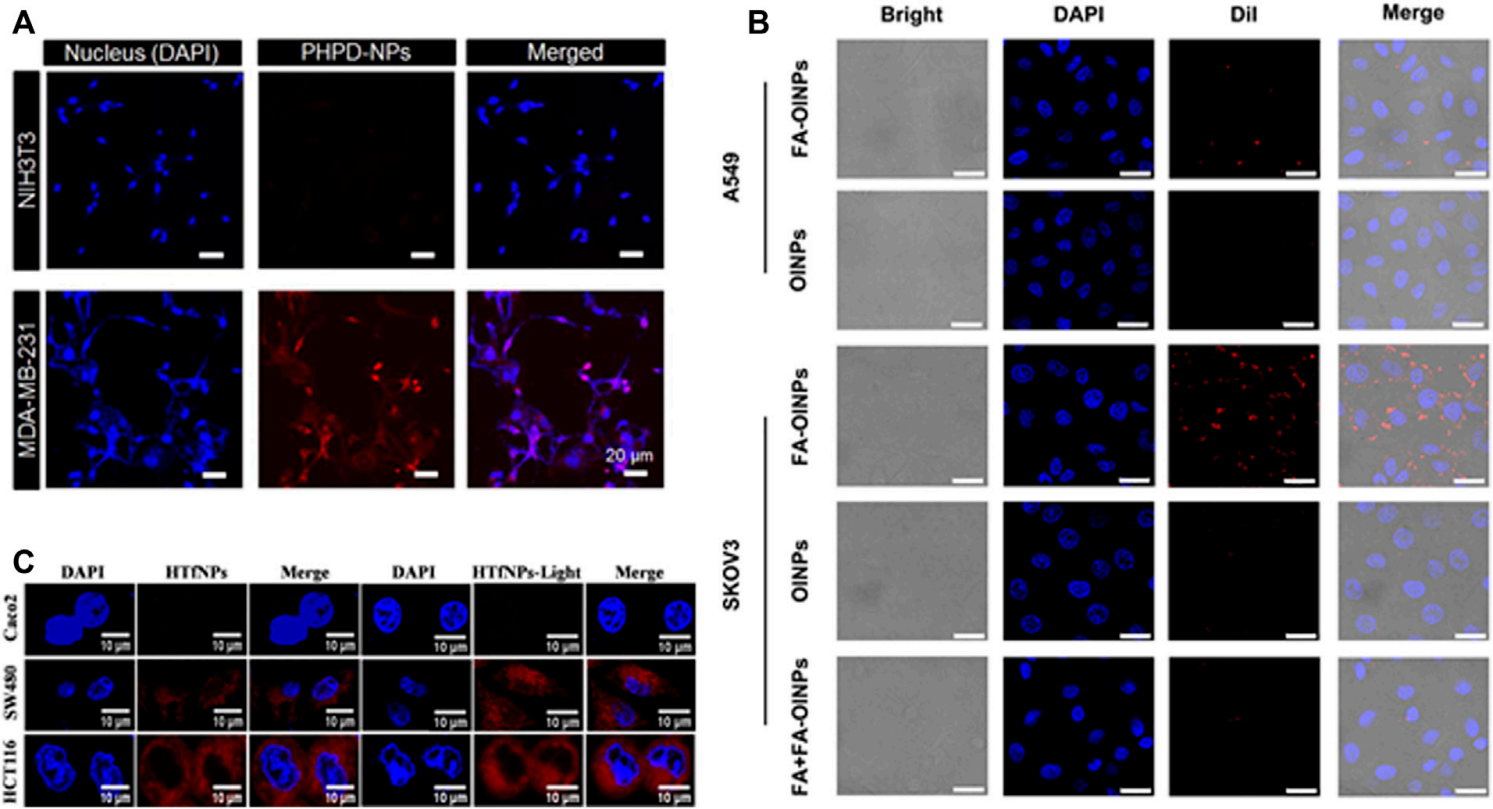
Schematic illustrations of confocal laser scanning microscopy images of different types of nanoparticles and their targeting efficiency with different receptors. **(A)** CD 44 receptor—reprinted with permission from reference (Han et al., 2016), Copyright 2016, American Chemical Society; **(B)** Folate receptor—reprinted with permission from reference (Liu et al., 2019), Copyright 2019, American Chemical Society and **(C)** Transferrin receptor—reprinted with permission from reference (Sardoiwala et al., 2020), Copyright 2020, American Chemical Society.

In cancer PDT research, nanocarrier-based targeted approaches show some promising results. Figure 4 shows schematic illustrations of confocal laser scanning microscopy images of various types of nanoparticles and their targeting efficiency with different receptors.

Core-shell nanoparticles in the size range of ∼ 130 nm were synthesized to target CD 44 receptor on the MDA-MB 231 cell line, which is CD 44 receptor overexpressing cell line. A conventional small-molecule photosensitizer (PS) loaded polydopamine nanoparticles serve as a shell, and PS conjugated hyaluronic acid serves as a shell molecule. An *in vitro* cancer-targeting efficiency of synthesized core-shell nanoparticles was tested with both CD44 high expressing (MDA-MB 231 cells) and CD44 low expressing cells (NIH3T3 cells) using confocal laser scanning microscopy. Because of the presence of hyaluronic acid on the shell, the fluorescence signals in MDA-MB 231 cells were significantly higher than in NIH3T3 cells (Han et al., 2016). Lipid-based nanoparticles are used to target folate receptors (FRs) overexpressing SKOV3 cell line (human ovarian cancer cells), the synthesized nanoparticles are in the size range of 301.08 ± 11.12 nm. Multifunctional theranostic nanoparticles (folate-targeted and oxygen/indocyanine green-loaded lipid nanoparticles) are synthesized using a two-step emulsion method. The confocal microscopy study found that folic acid (FA) loaded nanoparticles treated SKOV3 cells had a stronger fluorescence signal than A549 cells. A quantitative flow cytometry study using SKOV3 treated cells revealed that the fluorescence intensity of FA conjugated lipid nanoparticles was 2.3 times higher than that of FA unloaded nanoparticles (Liu et al., 2019).

Sardoiwala et al. (2020) synthesized hypericin-loaded transferrin nanoparticles with the size range of ∼65 ± 5 nm and aimed to target transferrin receptor in three different colon cancer cells {Caco2 [low expression of CD71 (transferrin receptor)], SW480, and HCT116 cells [higher expression of CD71 (transferrin receptor)]}. Cellular internalization studies revealed that higher expression of synthesized nanoparticles in HCT116 and SW480 cells than in Caco2 cells, and this was confirmed with flow cytometric studies, and the observed percentages are higher in HCT116 (80–90%) and SW480 (70–80%) cells than Caco2 cells (10–20%) . Cai et al., synthesized dual-targeted organic nanoparticles with two targeting ligands: folate and cRGD peptide in different ratios. The nanoparticles are ∼ 20 nm in size and are designed to target both folate and αvβ3 integrin receptors. An *in vitro* cellular uptake study was performed with NIH/3T3 normal cells and U87MG glioblastoma cells. Surface density of 75% cRGD and 25% FA showed a better cellular uptake on U87MG glioblastoma cells than NIH/3T3 cells (Cai et al., 2019).

## 5 Future perspectives and conclusion

In this review article, we discussed the most recent advancements in the use of biocompatible nanoparticles for targeted PDT cancer therapy. We focused on three types of biocompatible nanoparticles: polymeric nanoparticles, liposomes and dendrimers. It demonstrates promising potential and distinct advantages on surface modification using various target ligands/antibodies for cancer therapy. The results of an *in vitro* and *in vivo* study confirmed the benefits of using those biocompatible nanocarriers for cancer PDT. Both passive and active targeting mechanisms show significant advantages on using biocompatible nanocarriers to deliver therapeutic drugs/photosensitizers with enhancing the accumulation of loaded compounds to the targeted site of action. Furthermore, we reported the most used targeting ligands and targeting receptors in nanomedicine for various types of targeted cancer therapy. The advancement of nanoengineering provides excellent opportunities in targeted cancer PDT for various types of cancers. Some advanced nanoengineering applications, such as dual receptor targeting strategies, intracellular organelle targeting, and multifunctional nanocarrier will have a significant impact on current treatment modalities.

## Abbreviations

BNs, Biocompatible nanocarriers; 4T1, Breast cancer cells; CD44, Cell surface adhesion receptor 44; Ce6, Chlorin e6; DNA, Deoxyribonucleic acid; EPR, Enhanced Permeation and Retention; FDA, Food and Drug Administration, FRs, Folate receptors; HER, Herceptin; ICG, Indocyanine green; MCF-7, Breast cancer cells; mg, Miligram; mTHPC, m-tetrahydroxyphenylchlorin; NIR, Nearinfrared; nm, Nanometer; PBS, Phosphate buffered saline; PCL, Polycaprolactone; PDT, Photodynamic therapy; PNIPAAM, Poly-N-isopropylacrylamide; PpIX, Protoporphyrin IX; PTT, Photothermal therapy; RB, Rosebengal; SiNc, Silicon naphthalocyanine; TBD, 1,5,7-triazabicylo [4.4.0] dec-5-ene; TPZ, Tirapazamine; WHO, World Health Organization.
